# Organelle Engineering in Yeast: Enhanced Production of Protopanaxadiol through Manipulation of Peroxisome Proliferation in *Saccharomyces cerevisiae*

**DOI:** 10.3390/microorganisms10030650

**Published:** 2022-03-18

**Authors:** Bo Hyun Choi, Hyun Joon Kang, Sun Chang Kim, Pyung Cheon Lee

**Affiliations:** 1 Department of Molecular Science and Technology, Ajou University, World Cup-ro, Yeongtong-gu, Suwon 16499, Korea; bohyunho0@ajou.ac.k (B.H.C.); hyunrlajkang@ajou.ac.kr (H.J.K.); 2 Department of Biological Sciences, Korea Advanced Institute of Science and Technology (KAIST), 291 Daehak-ro, Yuseong-gu, Daejeon 34141, Korea; sunkim@kaist.ac.kr

**Keywords:** ginsenosides, peroxisome, targeting, isoprenoids

## Abstract

Isoprenoids, which are natural compounds with diverse structures, possess several biological activities that are beneficial to humans. A major consideration in isoprenoid production in microbial hosts is that the accumulation of biosynthesized isoprenoid within intracellular membranes may impede balanced cell growth, which may consequently reduce the desired yield of the target isoprenoid. As a strategy to overcome this suggested limitation, we selected peroxisome membranes as depots for the additional storage of biosynthesized isoprenoids to facilitate increased isoprenoid production in *Saccharomyces cerevisiae*. To maximize the peroxisome membrane storage capacity of *S.cerevisiae*, the copy number and size of peroxisomes were increased through genetic engineering of the expression of three peroxisome biogenesis-related peroxins (Pex11p, Pex34p, and Atg36p). The genetically enlarged and high copied peroxisomes in *S.cerevisiae* were stably maintained under a bioreactor fermentation condition. The peroxisome-engineered *S.cerevisiae* strains were then utilized as host strains for metabolic engineering of heterologous protopanaxadiol pathway. The yields of protopanaxadiol from the engineered peroxisome strains were ca 78% higher than those of the parent strain, which strongly supports the rationale for harnessing the storage capacity of the peroxisome membrane to accommodate the biosynthesized compounds. Consequently, this study presents in-depth knowledge on peroxisome biogenesis engineering in *S.cerevisiae* and could serve as basic information for improvement in ginsenosides production and as a potential platform to be utilized for other isoprenoids.

## 1. Introduction

Owing to the increasing demand for natural resources, environment friendly biosustainable processes are needed to limit the overuse of fossil fuels and address mounting environmental issues. These changes in the social and economic paradigm and the rapid development of engineering and analytical techniques have promoted the application of microbial metabolic engineering to diverse fields, such as food, pharmaceutical, cosmetic, biochemical, and biofuel industries. The reports of successful commercial microbial processes for the production of biofuels, natural or non-natural chemicals, and pharmaceuticals have been published consistently [1,2]. The range of products obtained via microbial processes can be expanded by merging synthetic biology and bioinformatics with microbial metabolic engineering [3,4].

Isoprenoids are natural compounds with diverse structures and are derived from the precursor isopentenyl diphosphate (IPP), which is synthesized through two distinct pathways: the 2-C-methyl-D-erythritol 4-phosphate (MEP) pathway or the mevalonate (MVA) pathway. Most eukaryotes, including yeasts, and some prokaryotes, such as *Streptomyces*, *Flavobacterium*, and *Staphylococcus*, use the MVA pathway for IPP biosynthesis, whereas most prokaryotes use the MEP pathway for the same [5,6]. The mass production of isoprenoids associated with biological activities beneficial to humans is being undertaken increasingly because of the high value of isoprenoids in the biotechnology industry [7]. However, the increasing demand for isoprenoids exceeds the supply of plant-derived isoprenoids (primarily owing to seasonal variations and relatively poor yield and purification) or chemically synthesized isoprenoids (primarily owing to the challenges of synthesizing the highly complex chemical structures) [8]. Microorganisms are excellent alternatives to plants as sources of novel biochemical compounds, as their secondary metabolic pathways are tremendously diverse [9]. The rapid development of metabolic engineering, synthetic biology techniques, and bioinformatics have significantly improved the systems-level understanding of metabolic alterations in host microorganisms including *Escherichia coli* and *Saccharomyces cerevisiae* [10].

Yeast strains have been utilized widely and for a long time for the production of natural compounds [11]. Thorough knowledge of the genetics and cellular metabolism of yeast, ease of genetic manipulation, and solid resistance in various environments are major factors that affect the use of yeast as a microbial host in bioprocesses [12]. In contrast to bacteria, yeasts have unique subcellular organelles (such as mitochondria, endoplasmic reticulum (ER), Golgi apparatus, peroxisomes, and vacuoles) and targeting systems for the accurate localization of proteins into specific organelles via well-characterized signals [13]. The metabolic engineering of yeast strains involves the reconstruction and expression of heterogeneous pathways while considering the efficiency of the spatial optimization of metabolic pathways [14]. Specific properties of subcellular organelles can be used to facilitate compartmentalization by targeting metabolic pathway proteins for the production of target compounds; for example, the mitochondria can be used for producing isoprene [15], amorphadiene [16], and hydrocortisone [17]; the peroxisomes can be used for producing penicillin [18], alkanes [19], polyhydroxyalkanoates [20], and squalene [21]; the ER can be used for producing opioids [22]; and vacuoles can be used for producing methyl halides [23]. The compartmentalization of metabolic pathways is advantageous as it helps use the unique environment of each organelle for target compound biosynthesis and the distinct physiochemical properties and metabolites (or precursors) typically produced in each organelle for maintaining desirable pathway flux balances [24].

In addition to the subcellular organelles that can be used advantageously, organelle membranes have the potential to provide additional storage space for the accumulation of heterogeneous hydrophobic target compounds. The subcellular membrane, which provides additional storage space, can be used in synthetic biology experiments as a material for maximizing the production of hydrophobic compounds in microbial hosts, as its use would help alleviate the inherent negative effects of stored hydrophobic compounds on the function and integrity of cellular membranes in microorganisms. In particular, physiologically inducible subcellular organelles, such as peroxisomes [25,26], are good model systems for evaluating the utility of subcellular organelle membranes as additional cellular storage space for maximizing the yields of target compounds in eukaryotic microbial hosts. Although a recent study reported utilizing peroxisomes in *S.cerevisiae* strains [27], there are few reports on genetic engineering in peroxisome biogenesis (such as manipulation of size and copy number of peroxisomes) and its exploration for heterologous pathway engineering.

Therefore, as a proof-of-concept study, we genetically engineered the peroxisome biogenesis of *S.cerevisiae* by modulating the expression of three peroxisome proliferation-related proteins (PEX). The constructed peroxisome-proliferated *S.cerevisiae* strains stably maintained the enlarged sizes and/or high copy numbers of peroxisomes, which can serve as an additional storage membrane for biosynthesized isoprenoids. In these engineered strains, the heterologous protopanaxadiol [28] biosynthetic pathway was induced to evaluate whether the expanded membranes of the engineered peroxisomes could be used for additional storage of protopanaxadiol.

## 2. Materials and Methods

### 2.1. Strains and Media

The *S.cerevisiae* strains used in this study are listed in Table 1. The CEN PK2-1D strain was used as the parent strain for the construction of engineered *S.cerevisiae* strains. *S.cerevisiae* strains were aerobically cultured in 250 mL baffled flasks containing the YPD medium (10 g/L yeast, 20 g/L peptone, and 20 g/L glucose) at 30 °C with shaking at 250 rpm. The complex medium YPDO (10 g/L yeast, 20 g/L peptone, 10 g/L glucose, 10 g/L oleic acid, and 1 g/L Tween 40) was also used to evaluate peroxisome biogenesis in the engineered strains. The SD medium (6.7 g/L yeast nitrogen base (Difco, Sparks, MD, USA), 20 g/L glucose, and a mixture of amino acids and nucleotides except leucine, uracil, tryptophan, or histidine (synthetic drop-out medium supplements, (Sigma Aldrich, Saint Louis, MO, USA)) was used as a selective medium for the engineered autotrophic strains. Cell growth was monitored by measuring an optical density at 600 nm (OD_600_) on a SpectraMax^®^ Plus384 spectrophotometer (Molecular Devices, San Jose, CA, USA). *Escherichia coli* XL1-Blue strain, which was used for the construction and propagation of plasmids (Table 1), was cultivated in Luria broth supplemented with 100 mg/L ampicillin at 37 °C with shaking at 250 rpm.

### 2.2. Plasmid Construction

The plasmids and PCR primers used in this study are listed in Table 2 and Supplementary Table S1, respectively. The genomic DNA of *S.cerevisiae* and *X. dendrorhous* was isolated using the MG Cell Genomic DNA Extraction SV Miniprep kit (MGmed, Seoul, South Korea). Gene-specific PCR primers were designed based on the corresponding gene sequences from the GenBank database (https://www.ncbi.nlm.nih.gov/genbank/). To manipulate peroxisome biogenesis, two genes encoding peroxisomal membrane protein 34 (PEX34) and ADH2 from *S.cerevisiae* were amplified using PCR with gene-specific primers constructed from the genomic DNA of *S.cerevisiae* and then subcloned into pUC57_URAblast and pCEV-TEF1, respectively, thereby constructing pUC57_URAblast_PEX34 and pCEV-TEF1-ADH2, respectively. Two genes encoding EGFP and monomeric Kusabira-Orange fluorescent protein (mKO) were amplified using PCR with specific gene primers from pRS424-GPD-EGFP and pRS425-GPD-mKO, respectively, and used as fusion fluorescent partner genes to construct fusion fluorescent proteins. To construct expression modules for the dammarenediol II and protopanaxadiol synthesis pathway proteins, ERG1, DS, PPDS, and CPR (encoding squalene epoxidase (GenBank: AB003516.1), dammarenediol II synthase (GenBank: JN596111.1), protopanaxadiol synthase (GenBank: DI172794.2), and cytochrome 450 reductase (GenBank: KF486915.1) from *P. ginseng* were chemically synthesized with codon optimization for *S.cerevisiae* from MUTAGENEX (Columbus, OH, USA). The four synthesized pathway genes were subcloned into expression vectors to construct pRS426-PGK1-ERG1pg, pCEV-G1-TEF1-DS, pCEV-G1-TEF1-CPR, and pCEV-G1-PGK1-PDS. Additionally, ERG9, encoding squalene synthase, and tHMG1, encoding truncated HMG-CoA reductase, were amplified using PCR with gene-specific primers from the gDNA of *S.cerevisiae* and subcloned into expression vectors, thereby generating pRS424-GPD-ERG9 and pCEV-TEF1-tHMG1, respectively. Individual genes, along with the promoter and terminator regions, were amplified using PCR from a corresponding expression plasmid and then subcloned into YIplac128, YIplac204, YIplac211, or pUC57-URAblast (an integrative plasmid).

### 2.3. Strain Construction

Standard techniques and culture media were used for the genetic modification of *S.cerevisiae* strains (Table 1). Genome editing in *S.cerevisiae* strains was performed using general marker-based homologous recombination selection. Plasmids for genome editing were constructed using the general restriction enzyme-based technique. DNA fragments for genome integration or gene deletion were constructed using overlapping PCR and were used to transform *S.cerevisiae* strains using the standard lithium-acetate method. The transformants were then subjected to selective SD agar plating. Marker-free genome-edited *S.cerevisiae* strains were constructed based on the URA-blaster and 3Myc fragment system. The genome-edited regions were confirmed using Sanger sequencing or PCR.

### 2.4. Isolation of Peroxisomes and Quantification of Proteins Present in the Isolated Peroxisomes

Subcellular fractionation and peroxisome isolation were performed using the Optiprep Peroxisome Isolation Kit (Sigma-Aldrich, Saint Louis, MO, USA). Cells grown to an OD_600_ of 0.7 in YPD medium were harvested and converted into spheroplasts by digestion with 20 U zymolyase (ZYMO Research, Irvine, CA, USA) for 90 min by occasionally measuring the OD_600_ and observing under a microscope. Using a buffer (1.2 M sorbitol, 0.1 M K_2_HPO_4_, pH 7.5) containing 1× complete protease inhibitor cocktail (Roche, Ludwigsburg, Germany), spheroplasts were homogenized using a glass dounce homogenizer with 5–23 gentle strokes. The homogenate was first centrifuged for 10 min at 1000× *g* and then further centrifuged for 10 min at 2000× *g* to collect the post-nuclear supernatant fraction. The post-nuclear supernatant fraction was then subjected to differential centrifugation at 25,000× *g* for 30 min to collect the crude peroxisomal fraction. The crude peroxisomal fraction was then applied to density gradient separation with the Optiprep Peroxisome Isolation Kit (Sigma-Aldrich, St. Louis, MO, USA) with 100,000× *g* ultracentrifugation at 4 °C for 9 h. The peroxisomal fraction (2 mL) was collected from the bottom layer of the gradient. The concentration of total protein in the peroxisomal fraction was measured using the Bradford method with bovine serum albumin as the standard [29].

### 2.5. Analysis of the Sensitivity of Strains to Oxidative Stress

To determine the survival of peroxisome-engineered and WT yeast cells under oxidative stress, a method reported by Liu et al. [30] was used with minor modifications. Yeast cells collected at an OD_600_ of 1.0 were harvested, washed with sterile water, and suspended at a final density of 1 × 10^8^ cells/mL in 100 mM phosphate buffer (pH 6.5). Then, the same volume of H_2_O_2_ was added at different concentrations (0, 0.5, and 1 mM) to the suspension, and the treated yeast cells were incubated at 30 °C in a shaker at 200 rpm for 30 min. The incubated yeast cells were collected by centrifugation and washed one time with sterile water, following which 5 μL of 10-fold serial dilutions (from 1 × 10^7^ to 1 × 10^4^ cells/mL) was plated on the solid YPD or SD medium at 30 °C. Colony formation on the plates was monitored after 48 h of incubation. The effect of supplementation with 10 mM ascorbic acid, 10 mM adenine [31], 10 mM cysteine, and 10 mM methionine on the survival of peroxisome-engineered and H_2_O_2_-treated yeast strains was determined using the method described above. To evaluate the mitochondrial membrane potential, MitoTracker Red CMXRos (Thermo Fisher Scientific, Waltham, MA, USA) was added directly to a 500 μL exponential phase (OD_600_ of 1.0) culture in the SD medium to a final concentration of 2.5 mM. Yeast cells were incubated at 30 °C for 30 min, washed with sterile water, and observed immediately using a fluorescence microscope. To confirm the more pronounced changes in the mitochondria in peroxisome-engineered and WT strains, EGFP was linked at the C-terminus of tetratricopeptide repeat protein 70 (TOM70) and soluble F-box protein Mfb1, which are mitochondrial membrane proteins. TOM70-EGFP- or Mfb1-EGFP-expressing strains were cultured in the SD medium to an OD_600_ of 1.0 at 30 °C, collected, washed with sterile water, and observed using fluorescence microscopy (see Section 2.7 for details).

### 2.6. Bioreactor Fermentation

Batch fermentation was performed at 30 °C, pH 5.5, and a dissolved oxygen (DO) level >50% in a 3.5 L BioFlo 320 bioreactor (Eppendorf, Hamburg, Germany) containing 1.5 L of the SD, YPD, or YPDO medium. The DO level was maintained by automatically increasing the agitation rate from 300 to 500 rpm and supplying pure O_2_ gas at a flow rate of 1.5 vol/vol/min (vvm). The pH was controlled at 5.5 by the automatic addition of 8% (*v*/*v*) NH_4_OH and 2 N HCl solutions. The concentrations of glucose and ethanol were measured using an Agilent 1200 HPLC equipped with an Agilent 1200 refractive index detector and an Aminex^®^ HPX-87H column (7.8 × 300 mm, Bio-Rad, Hercules, CA, USA) at a flow rate of 0.7 mL/min, using 4 mM H_2_SO_4_ as the isocratic mobile phase.

### 2.7. Fluorescence Microscopy and Image Processing

Engineered *S.cerevisiae* strains expressing GFP and/or mKO fusion proteins were aerobically cultured in a 500 mL baffled flask containing 100 mL of the SD medium for 16 h, in 100 mL of the YPD or YPDO medium for 144 h. After 1 mL of the culture was collected periodically, the cells were washed twice with 1 mL of 0.5× PBS buffer, resuspended in 450 μL of 0.1 M potassium phosphate, fixed by adding 50 μL of the formaldehyde solution, and incubated at 30 °C for 30 min. The fixed cells (5 μL) were immediately visualized using a LEICA DM 2500 microscope (LEICA, Solms, Germany) equipped with a LEICA DFC450 C digital camera and FITC filter set. The GFP signal was visualized using a 470/40 nm (excitation) and 525/50 nm (emission) filter set at an exposure time of 700 ms. The mKO fluorescence signal was visualized using a 546/12 nm (excitation) and 575/40 nm (emission) filter set at an exposure time of 700 ms. The images were resized and cropped using the Adobe Photoshop program without further processing.

### 2.8. FACS Analysis

FACS analysis was conducted using a BD FACSCalibur flow cytometer (BD Biosciences, Franklin Lake, NJ, USA). Five milliliters of engineered *S.cerevisiae* cells expressing GFP were pelleted, washed twice with 10 mL of 1× PBS buffer, and resuspended in 1 mL of 1× PBS buffer. The GFP signal from 100,000 cells was obtained by excitation with a 488 nm argon ion laser (15 mW) and detection with a 530/30 nm band-pass filter. The data were analyzed using BD FACSDiva software (version 5.0, BD Biosciences, Franklin Lake, NJ, USA).

### 2.9. TEM Analysis

TEM analysis was conducted at the NICEM facility (Seoul National University, Seoul, South Korea). Engineered cells were cultured in 100 mL of the SD medium to the mid-log growth phase, pelleted, washed twice with 40 mM potassium phosphate, pelleted, and fixed using 1.5 mL of the fixing solution (1.25% formaldehyde, 2.5% glutaraldehyde, and 40 mM potassium phosphate, pH adjusted to 7.0) for 20 min at room temperature. The cells were further treated with 2% KMnO_4_, embedded, cut with a diamond knife on an ultramicrotome (EM UC7, LEICA, Wetzlar, Germany), and collected on 200 mesh copper grids (Electron Microscopy Sciences, Hatfield, PA, USA). After staining with uranyl acetate and lead citrate, the cells were visualized using a LIBRA 120 energy-filtering transmission electron microscope (Carl Zeiss, Oberkochen, Germany) at 120 kV. Images were recorded using a bottom-mounted 2 k Å~2 k CCD camera.

### 2.10. Extraction and Quantification of Dammarenediol II and Protopanaxadiol

For the extraction of dammarenediol II and protopanaxadiol, 10 mL of cell culture was harvested by centrifugation at 4000 rpm and 4 °C for 30 min. Next, 700 μL of a mixture of acetone and methanol (1:1, *v*/*v*) and ca. 20 glass beads (acid-washed, dimension ~5 mm, Sigma Aldrich) were added to the cell pellet. The cell suspension was physically agitated in a Precellys homogenizer (Bertin Technologies, Fontaine, France) with an operating option of three cycles (1 min beating, 30 s holding). After centrifugation at 13,000 rpm for 10 min, the collected organic extract was filtered using a GHP membrane filter (0.45 µm, Pall) for analysis. Ten microliters of the extract containing dammarenediol II and/or protopanaxadiol was added to a Zorbax eclipse XDB-C18 column (4.6 × 150 mm, 5.0 μm; Agilent Technologies, Santa Clara, CA, USA) and eluted under gradient conditions with a solvent system (acetonitrile/water) at a flow rate of 1 mL/min using an Agilent 1200 HPLC system equipped with a photodiode array detector (Agilent Technologies). The chromatogram was acquired at 203 nm. Quantification was performed based on authentic dammarenediol II and protopanaxadiol (Sigma Aldrich) as standards. The results are presented as mean values ± standard deviation (SD) from three independent measurements.

### 2.11. Statistical Analysis

Results are expressed as the mean ± standard deviation of three replicates (*n* = 3) in determination of protopanaxadiol concentration and peroxisomal protein quantification in each mutant strain and six replicates (*n* = 6) for counting peroxisome numbers and estimating sizes. Statistical analysis for peroxisomal protein and protopanaxadiol concentration were performed using one way ANOVA with SigmaPlot 12.0 (Systat Software Inc., San Jose, CA, USA). Values of * *p* < 0.5, ** *p* < 0.01, or *** *p* < 0.001 were considered statistically significant.

## 3. Results

### 3.1. Engineering of Peroxisome Proliferation in S.cerevisiae

**Figure 1:**
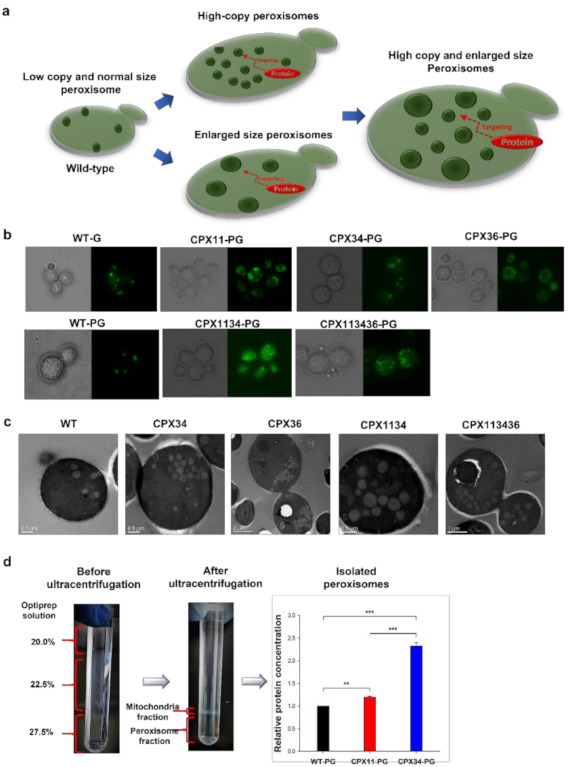
Engineering peroxisome biogenesis in *Saccharomyces cerevisiae*. (**a**) Schematic representation of the engineering of peroxisome biogenesis for increasing the copy number and size of peroxisomes in *S.cerevisiae*. (**b**) Fluorescence microscopy images of changes in the peroxisomes of peroxisome-engineered and wild-type (WT) strains. Genotype of the peroxisome-engineered strains are described in Table 1. Enhanced green fluorescent proteins targeted to peroxisomes were constructed by fusion with peroxisomal oxoacyl thiolase (PG) or peroxisome targeting signal 1 (PTS1) and then expressed in WT and peroxisome-engineered strains. (**c**) Transmission electron microscopy analysis of changes in the peroxisomes of peroxisome-engineered and WT strains. Scale bars: 0.5 μm or 1.0 μm. (**d**) Relative protein concentration of isolated peroxisomes from WT-PG, CPX11-PG, and CPX34-PG strains. Data are presented as the mean ± standard deviation of biological triplicates for protein quantification (*n* = 3) and six replicates for counting peroxisome numbers (*n* = 6) (** *p* < 0.01, *** *p* < 0.001).

One of the major considerations in optimizing isoprenoid production in microbial hosts is that isoprenoid accumulation within intracellular membranes may impede balanced cell growth, which may consequently reduce the desired yield of the target isoprenoid [32]. To overcome this suggested limitation, we selected peroxisomes to expand the membrane capacity and facilitate high accumulation of protopanaxadiol by genetically increasing the copy number and size of peroxisomes (Figure 1a). PEX interactions were analyzed to verify their functions in peroxisome biogenesis in *S.cerevisiae*, and many PEX proteins have been shown to be functionally involved in peroxisome biogenesis [33]. Among PEX proteins, Pex34p (peroxisome-population-regulated protein) [34], Pex11p (peroxisome-population-regulated protein) [35], and Atg36p (autophagy-related protein) [36] encoded by PEX34, PEX11, and ATG36, respectively, were selected to manipulate peroxisome biogenesis in *S.cerevisiae*. Three peroxisome-biogenesis-mutant strains (CPX34: PEX34-overexpressing mutant; CPX36: ATG36 null mutant; and CPX11: PEX11 null mutant) were developed by genome editing (see Strain Construction in Materials and Methods). Before investigating peroxisome biogenesis in the three mutants, the copy number and size of peroxisomes in wild-type (WT) *S.cerevisiae* were evaluated using enhanced green fluorescent protein (EGFP) fused to peroxisomal targeting signal 1 (PTS1) peptide or peroxisomal 3-ketoacyl-CoA thiolase (POT1) [37]. The EGFP fusion constructs targeting peroxisomes were designed to be expressed on a plasmid (as pRS424-GPD-EGFPp1), which generated the WT-G strain, and on the genome (as POT1-GFP), which generated the WT-PG strain. Fluorescence microscopy analysis showed that EGFP fused with POT1 or PTS1 localized to the peroxisomes of WT-G and WT-PG (Figure 1b). The peroxisomes were estimated to have a copy number of approximately 3.5 ± 1.4 per cell in WT-G and WT-PG (*n* = 6), which agreed well with the reported peroxisome copy number in WT *S.cerevisiae* [38,39]. Based on these results, genetically engineered peroxisome biogenesis in CPX34, CPX36, and CPX11 was investigated by expressing POT1-GFP on the genome of each mutant, and the strains were referred to as CPX34-PG, CPX36-PG, and CPX11-PG, respectively. PEX34 overexpression (CPX34-PG) and ATG36 deletion (CPX36-PG) increased the number of peroxisomes (ca. 16.3 ± 3.3 copies per CPX34 (*n* = 6, *p* < 0.001) or 13.5 ± 2.2 copies per CPX36 cell (*n* = 6, *p* < 0.001) vs. ca. 3.5 ± 1.4 (*n* = 6) copies per WT-G or WT-PG cell) but did not significantly alter the peroxisome size (Figure 1b,c).

Unlike PEX34 overexpression and ATG36 deletion, PEX11 deletion (which formed the CPX11-PG strain) did not significantly influence the copy number of peroxisomes but generated enlarged and clustered peroxisomes. These changes in peroxisome biogenesis in the three mutants agree well with the findings from previous studies [34,35,36]. To indirectly measure the degree of increase in peroxisomal membranes, peroxisomes were isolated from WT-PG, CPX11-PG (for peroxisomes with enlarged size), and CPX34-PG (for peroxisomes with increased copy number) strains. Analysis of relative protein concentration in the isolated peroxisomes revealed that CPX34-PG contained ca. 140% more protein than that in WT-PG, but unexpectedly, CPX11-PG contained ca. 20% more protein than that in WT-PG (Figure 1d). The relatively low protein content in CPX11-PG might be explained by the altered density of peroxisomes caused by enlarged sizes during ultracentrifugation. Nevertheless, these results strongly support that the peroxisomal membranes were increased and served as additional storage for hydrophobic compounds such as protopanaxadiol.

### 3.2. Sensitivity of Peroxisome-Engineered S.cerevisiae to Oxidative Stress

**Figure 2:**
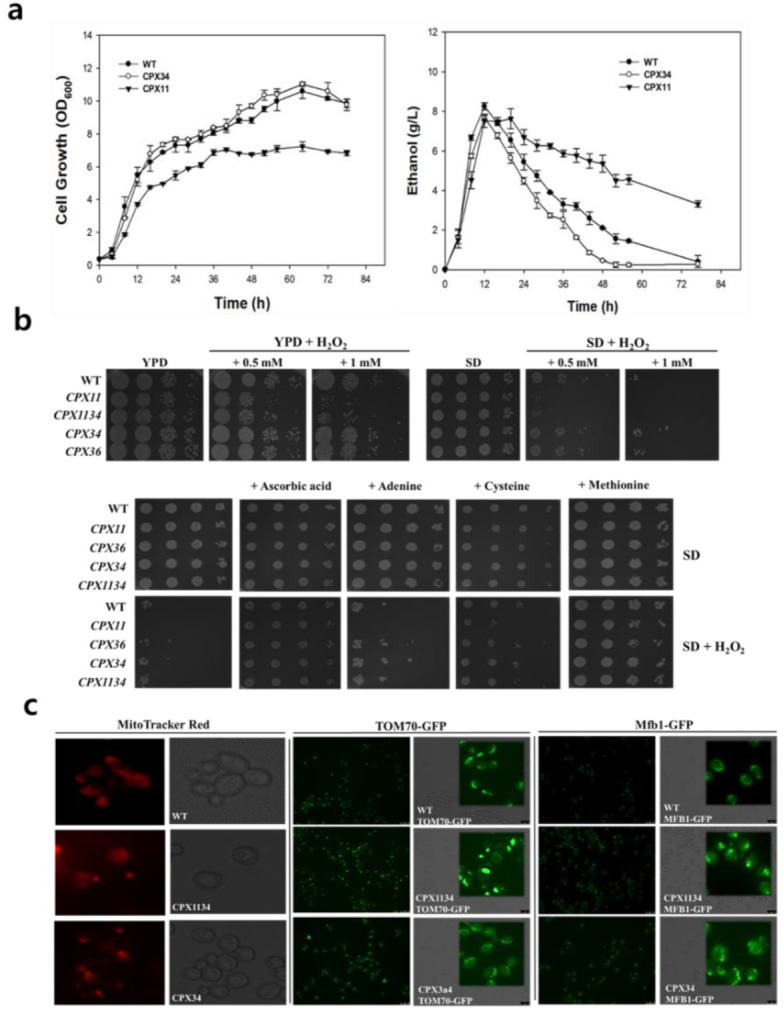
Sensitivity of peroxisome-engineered strains to oxidative stress. (**a**) Cell growth (**left**) and ethanol consumption (**right**) of wild-type (WT), CPX11, and CPX34 in YPD medium. Data are presented as the mean ± standard deviation of biological triplicates. (**b**) Growth of peroxisome-engineered strains (WT, CPX11, CPX34, CPX36, and CPX1134) on YPD and synthetic defined agar plates with and without 0.5 mM and 1 mM H_2_O_2_ supplementation (upper) and growth on synthetic defined agar plates with and without 1 mM H_2_O_2_ supplementation and cellular oxidative stress suppressors (ascorbic acid, adenine, cysteine, and methionine). (**c**) Fluorescent microscopy analysis of the mitochondrial morphology of WT, CPX34, and CPX1134. Fluorescence was monitored using fusion proteins of the mitochondrial proteins TOM70 and Mfb1 linked with enhanced green fluorescent protein and MitoTracker Red.

Under the liquid culture supplied with glucose, *S.cerevisiae* generally metabolizes glucose and produces ethanol in the medium. When glucose is depleted, the glucose metabolism is switched to ethanol respiratory metabolism (i.e., diauxic shift). Unexpectedly, the cell growth and ethanol consumption rate of the CPX11 strain were relatively slow compared to those of the WT or CPX34 strain (Figure 2). As peroxisome biogenesis is known to be influenced by cellular oxidative stress [40,41], the sensitivity of CPX11 to oxidative stress was investigated by monitoring the growth of CPX11 on yeast extract peptone dextrose (YPD) or complete synthetic defined (SD) agar under varying stress conditions. H_2_O_2_ supplementation significantly inhibited the growth of CPX11 on YPD and SD media, with high H_2_O_2_ concentration (0.5 mM) and defined medium (SD) inhibiting cell growth more severely than low H_2_O_2_ concentration (1 mM) and complex medium (YPD) (Figure 2a). The observed growth inhibition of CPX11 cells was relieved when four oxidative stress-reducing chemicals (ascorbic acid, adenine, cysteine, and methionine) [42,43] were added to the media (Figure 2b). Oxidative stress-related Pex11p-based peroxisome biogenesis in CPX11 may be related to the modified mitochondrial network (Figure 2c) that is frequently observed in cells under oxidative stress or starvation [44]. Collectively, these findings suggest that peroxin Pex11p is related to oxidative stress-responsive metabolism and peroxisome biogenesis, and the observed sensitivity of peroxisome-engineered strains to cellular oxidative stress could be relieved by the addition of oxidative stress-reducing chemicals into the medium.

### 3.3. Optimization of Peroxisome Proliferation in S.cerevisiae

**Figure 3:**
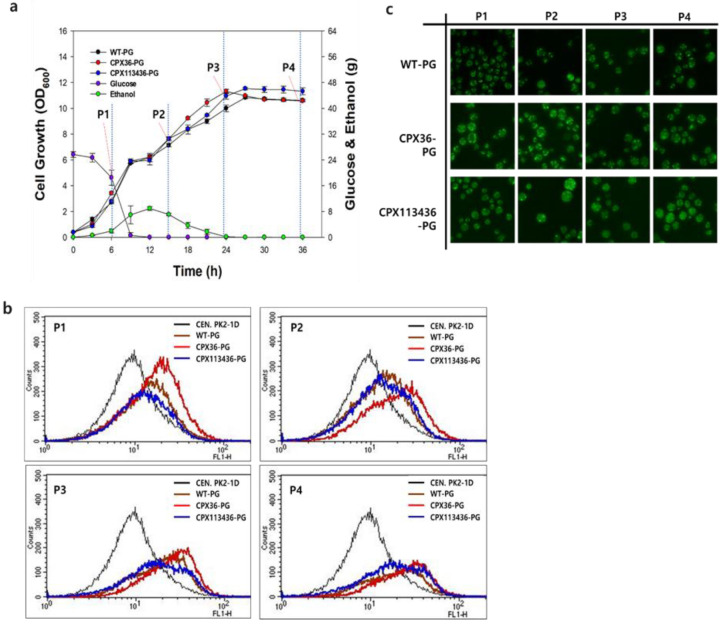
Peroxisome stability in peroxisome-engineered CPX36-PG and CPX113436-PG strains in a bioreactor. (**a**) Cell growth of WT-PG (low copy number: ca. 3.5 ± 1.4 copies per cell (*n* = 6)), CPX36-PG (medium copy number: ca. 13.5 ± 2.2 copies per cell (*n* = 6, *p* < 0.001)), and CPX113436-PG (high copy number: ca. 13.8 ± 3.1 copies per cell (*n* = 6, *p* < 0.001)) in four growth phases (P1: glucose consumption, P2: diauxic shift from glucose to ethanol, P3: post-diauxic shift, and P4: stationary growth phase). Data are presented as the mean ± standard deviation of biological triplicates. (**b**) FACS chromatograms of CEN.PK2-1D (WT strain without PG, a negative control), WT-PG (WT strain with PG), CPX36-PG, and CPX113436-PG in four growth phases. (**c**) Peroxisome profiles of WT-PG, CPX36-PG, and CPX113436-PG strains.

To increase the copy number and size of peroxisomes, two mutants were further generated by modulating the coexpression of PEX11, PEX34, or ATG36: the double mutant CPX1134 (PEX11 null and PEX34-overexpressing mutant) and the triple mutant CPX113436 (ATG36 null, PEX11 null, and PEX34-overexpressing mutant) (Table 1). Even though CPX1134 and CPX113436 have a PEX11-deleted genotype (CPX11), the cell growth and ethanol consumption rates of CPX1134 and CPX113436 were similar to those of the WT strain and greater than those of CPX11 (Figure 2b and Figure 3a), suggesting that the balance between the peroxisome copy number and size is a crucial factor in the engineering of peroxisome biogenesis. Next, CPX1134 and CPX113436 were engineered to express POT1-GFP on the genome of each mutant (named CPX1134-PG and CPX113436-PG, respectively), and the changes in peroxisome copy number and size were evaluated. Fluorescence microscopy and transmission electron microscopy (TEM) analyses revealed that the copy number and size of peroxisomes increased significantly in CPX1134 (and its fluorescent counterpart CPX1134-PG) and CPX-113436 (and its fluorescent counterpart CPX-113436-PG) (Figure 1b,c) compared to those in WT, CPX34, and CPX36 (copy number: ca. 10.1 ± 1.8 copies per CPX1134 cell (*n* = 6, *p* < 0.001) or ca. 13.8 ± 3.1 copies per CPX-113436 cell (*n* = 6, *p* < 0.001) vs. ca. 3.5 ± 1.4 (*n* = 6) copies per WT cell; size: ca. 350 ± 54.7 nm in CPX1134 (*n* = 6, *p* < 0.05) vs. ca. 333 ± 51.6 nm in CPX-113436 (*n* = 6, *p* < 0.05) vs. ca. 225 ± 41.8 nm in WT (*n* = 6)). Therefore, the higher copy number and enlarged peroxisomes in the engineered strains supported the successful proliferation of peroxisomes via the genetic manipulation of peroxisome biogenesis.

### 3.4. Peroxisome Stability in Engineered Strains

From a practical perspective, the high copy numbers and enlarged sizes of peroxisomes in the engineered strains need to be maintained during bioreactor cultivation for the stable production of target compounds. Peroxisome stability in the WT-PG (low copy number: ca. 3.5 ± 1.4 copies per cell (*n* = 6)), CPX36-PG (medium copy number: ca. 13.5 ± 2.2 copies per cell (*n* = 6, *p* < 0.001)), and CPX113436-PG (high copy number: ca. 13.8 ± 3.1 copies per cell (*n* = 6, *p* < 0.001)) strains was investigated by monitoring fluorescent peroxisomes in four growth phases (glucose consumption, diauxic shift from glucose to ethanol, post-diauxic shift, and stationary growth phase) for 36 h during a bioreactor batch fermentation in the YPD medium. Similar to that observed in flask cultivation, there was no inhibitory effect of high copy number and enlargement of peroxisomes on cell growth in the three strains during bioreactor batch cultures (Figure 3a). Fluorescence-activated cell sorting (FACS) chromatograms for all three strains rapidly shifted toward the right (higher intensity) until the starvation growth phase (Figure 3b), indicating the significant proliferation of peroxisomes in the strains.

Notably, the FACS chromatogram of CPX113436-PG broadened compared to those of WT-PG and CPX36-PG during the post-diauxic shift phase, indicating a more heterogeneous distribution of peroxisomes (diverse size and number of peroxisomes) in CPX113436-PG. The right-forward shifts in FACS chromatograms owing to significant changes in the peroxisomes were further confirmed by fluorescence microscopy analysis: the number and size of peroxisomes in CPX36-PG and CPX113436-PG strains started increasing during the glucose consumption phase and were maintained until the starvation growth phase (Figure 3c). Consequently, based on fluorescence microscopy and FACS analysis, peroxisomes exhibiting high proliferation (from medium to high copy number) in the engineered strains were stably maintained in the four growth phases during batch bioreactor cultivation.

### 3.5. Effect of Oleic Acid Supplementation on Peroxisome Biogenesis in Engineered Strains

**Figure 4:**
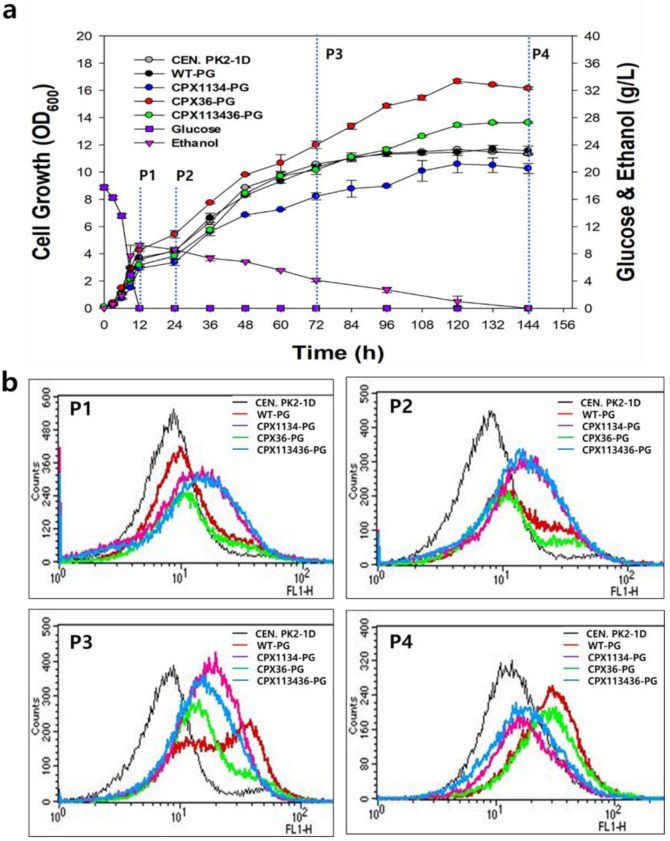
Effect of oleic acid supplementation on peroxisome biogenesis in peroxisome-engineered CPX36-PG, CPX1134-PG, and CPX113436-PG strains in a bioreactor. (**a**) Cell growth of CEN.PK2-1D (wild-type [WT] strain), WT-PG (WT strain with PG), CPX36-PG, CPX1134-PG, and CPX113436-PG in four growth phases (P1: glucose consumption, P2: diauxic shift from glucose to ethanol, P3: post-diauxic shift, and P4: starvation growth phase). Data are presented as the mean ± standard deviation of biological triplicates. (**b**) FACS chromatograms of CEN.PK2-1D (a negative control), WT-PG, CPX36-PG, CPX1134-PG, and CPX113436-PG in four growth phases.

Next, we supplied oleic acid, a peroxisome inducer [45,46], in bioreactor batch fermentation to investigate whether the proliferation of peroxisomes, which occurred at high levels in the engineered strains, was maximized to the cellular upper limit. The growth rate of CPX36-PG cells was highest in the YPD medium supplemented with oleic acid (named YPDO), followed by that of CPX113436-PG and WT-PG cells (and the non-fluorescent WT cells) (Figure 4a).

Notably, similar to the growth of the CPX11 strain, the growth of the CPX1134 strain was inhibited in the YPDO medium, suggesting that the oleic acid-induced enlargement of peroxisomes made the cells sensitive to oxidative stress in a manner similar to that observed in CPX11. FACS and fluorescence microscopy analysis revealed that oleic acid supplementation in the medium further increased the copy number and size of peroxisomes in WT-PG, CPX36-PG, and CPX113436-PG strains (Figure 4b), suggesting the greater potential for the modification of peroxisome biogenesis in peroxisome-engineered CPX36 and CPX113436 strains. However, because an extremely high peroxisome copy number and size would be a physiologically unfavorable to cells, the balancing of peroxisome proliferation and cell growth should be considered during the production of target compounds from peroxisome-engineered strains. As many PEX proteins involved in peroxisome biogenesis [33] have been reported, some PEX proteins could be utilized to genetically balance peroxisome biogenesis and cell growth in *S.cerevisiae*.

### 3.6. Construction of the Protopanaxadiol Pathway in Peroxisome-Engineered Strains

**Figure 5:**
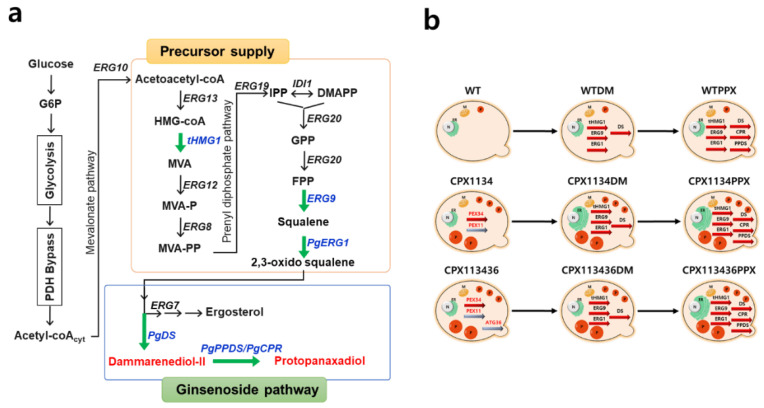
Production of dammarenediol II and protopanaxadiol via the mevalonate (MVA) pathway and schematic representation of the synthetic pathways in the engineered strains used in this study. (**a**) The black letters represent homogeneous enzymes, blue letters represent synthetic expression modules, and red letters represent target materials used in this study. The black arrows represent homogeneous pathway and green arrows represent heterogeneous pathways. The gene symbols and enzymes encoded by the genes (all heterogeneous genes of ginsenoside synthesis pathway were isolated from *Panax ginseng*, except where mentioned otherwise): ERG13, HMG-CoA synthase; tHGM1, truncated HMG-CoA reductase from *Saccharomyces cerevisiae*; ERG12, mevalonate kinase; ERG8, phosphomevalonate kinase; ERG19, mevalonate pyrophosphate decarboxylase; IDI1, IPP isomerase; ERG20, farnesyl pyrophosphate synthase; ERG9, squalene synthase from *S.cerevisiae*; PgERG1, squalene monooxygenase from *P. ginseng*; ERG7, lanosterol synthase; PgDS, dammarenediol II synthase; PgPPDS, protopanaxadiol synthase; PgCPR, cytochrome P450 reductase. Pathway intermediates: G6P, glucose-6-phosphate; HMG-CoA, 3-hydroxy-3-methylglutaryl coenzyme A; MVA, mevalonate; MVA-P, mevalonate-5-phosphate; MVA-PP, mevalonate pyrophosphate; IPP, isopentenyl pyrophosphate; DMAPP, dimethylallyl pyrophosphate; GPP, geranyl pyrophosphate; FPP, farnesyl pyrophosphate; (**b**) Reconstruction of ginsenoside biosynthetic pathway in the peroxisome-engineered strains (Table 1).

To evaluate the suitability of peroxisome-engineered *S.cerevisiae* strains for isoprenoid production, protopanaxadiol biosynthetic pathway (Figure 5a) was reconstructed in the peroxisome-engineered strains.

**Figure 6:**
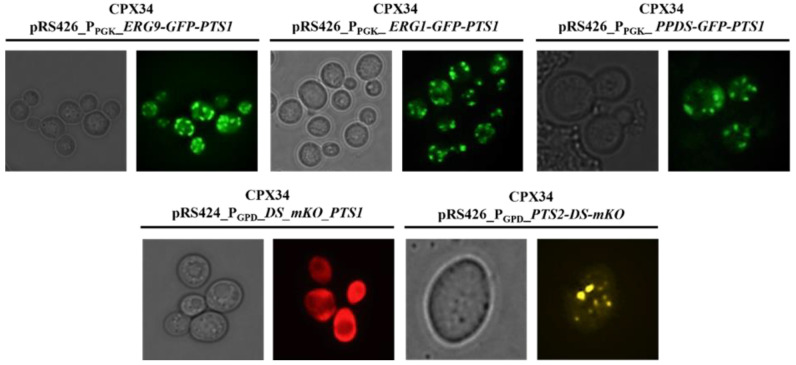
Fluorescence microscopy images of CPX34 strains engineered to target pathway enzymes into the peroxisomes. Peroxisome-engineered CPX34 cells harboring ERG9-GFP-PTS1, ERG1-GFP-PTS1, DS-mKO-PTS1, PTS2-DS-mKO, or PPDS-GFP-PTS1 fusion protein were cultured in the synthetic defined medium with 2% glucose for 12 h and then subjected to fluorescence microscopy.

As heterogeneously expressed proteins tend to randomly localize inside a cell [47], we first investigated whether two heterologous pathway proteins (DS and PPDS) from *Panax ginseng* and two endogenous precursor pathway proteins (EGR1 and ERG9) from *S.cerevisiae* could localize in peroxisomes to investigate the proper channeling of pathway intermediates in the CPX34 strain (medium copy number: ca. 16.3 ± 3.3 copies per cell (*n* = 6, *p* < 0.001)). Many PEX proteins are known to be programmatically transported to the peroxisome by PTS1 and peroxisomal targeting signal 2 (PTS2) [48,49]. Therefore, four fusion proteins in the order (pathway protein-fluorescent protein-PTS1) were designed to monitor the localization of pathway enzymes and construct the pRS424-GPD plasmid, thereby generating DS-mKO-pts1, PPDS-GFP-pts1, ERG1-GFP-pts1, and ERG9-GFP-pts1. In the construction of the four fusion proteins, PTS1 was preferentially selected because the peptide chain of PTS1 is shorter than that of PTS2 and can be fused at the C-terminus of fluorescent proteins. Florescence microscopy analysis (Figure 6) revealed that most of the expressed PPDS-GFP-pts1, ERG1-GFP-pts1, and ERG9-GFP-pts1, except for DS-mKO-pts1, was localized to the peroxisomes. DS-mKO-pts1 spread randomly inside cells without the specific targeting of peroxisomes by PTS1. The non-specific targeting of DS was overcome by using PTS2 (pts2-DS-mKO) instead of PTS1 (DS-mKO-pts1). Although the functional difference between PTS1 and PTS2 is yet to be clarified [50], PTS2 contains more hydrophobic amino acid residues than PTS1, which probably drives the DS of several transmembrane domains [51] into peroxisomes.

**Figure 7:**
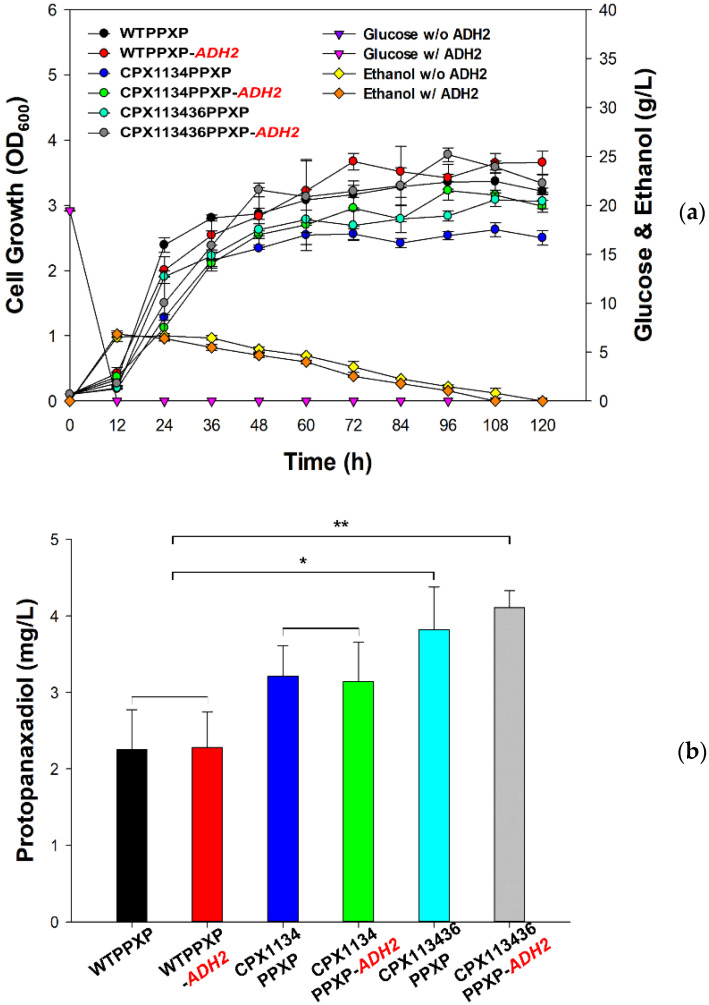
Cell growth and protopanaxadiol production in peroxisome-engineered and wild-type strains. (**a**) Cell growth of protopanaxadiol-producing CPX1134PPXP(pRS426), CPX1134PPXP(pRS426-ADH2), CPX113436PPXP(pRS426), CPX113436PPXP(pRS426-ADH2), WTPPXP(pRS426), and WTPPXP (pRS426-ADH2) strains cultured in synthetic defined medium containing 2% glucose for 120 h. (**b**) Protopanaxadiol production by CPX1134PPXP(pRS426), CPX1134PPXP(pRS426-ADH2), CPX113436PPXP(pRS426), CPX113436PPXP(pRS426-ADH2), WTPPXP(pRS426), and WTPPXP (pRS426-ADH2) strains at 120 h. Data are presented as the mean ± standard deviation of biological triplicates (* *p* < 0.05, ** *p* < 0.01).

After confirming the successful targeting of the two pathways and two precursor proteins into peroxisomes, as described above, the effect of peroxisome-targeting of pathway proteins with the help of a PTS system was investigated by measuring dammarenediol II production, which is an intermediate compound in the protopanaxadiol pathway (Figure 5a). The CPX1134DM (DM stands for dammarenediol II in CPX1134) and CPX1134DMP (DMP stands for dammarenediol II with peroxisome-targeting in CPX1134) strains produced dammarenediol II at levels approximately two times higher (6.1 ± 0.3 and 6.6 ± 0.6 mg/L, respectively) than those produced by the WTDM and WTDMP strains (3.2 ± 0.2 mg/L and 3.0 ± 0.1 mg/L, respectively). Based on the results of the observed positive effect of peroxisome-targeting with PTS, we next compared the production of protopanaxadiol by expressing the pathway proteins tagged with PTS (Table 2) in the genomes of the WT, CPX1134, and CPX113436 strains. Three protopanaxadiol-producing strains (Figure 5b) were constructed and named as WTPPXP (PPXP stands for protopanaxadiol with peroxisome-targeting in WT), CPX1134PPXP, and CPX113436PPXP (Table 1). In addition, overexpression of alcohol dehydrogenase 2 (ADH2) [52] by *S.cerevisiae* in the pRS426 plasmid was designed to promote ethanol consumption and cell growth of the three protopanaxadiol-producing strains, thus constructing three ADH2-coexpressing strains: WTPPXP(pRS426-ADH2), CPX1134PPXP(pRS426-ADH2), and CPX113436PPXP(pRS426-ADH2). Although ADH2 overexpression marginally enhanced ethanol consumption (Figure 7a), a moderate recovery (by approximately 20–30%) in the growth of two ADH2-coexpressing strains (CPX1134PPXP(pRS426-ADH2) and CPX113436PPXP(pRS426-ADH2)) was observed as they grew similarly to the WTPPXP and WTPPXP(pRS426-ADH2) strains in SD media.

However, the recovered growth of the two ADH2-coexpressing strains was still lower than the normal growth of WT in YPD media (Figure 2a). This indicates that additional manipulations are necessary, such as co-overexpression of heme activator protein 1 (HAP1) [53] to enhance ethanol consumption or medium optimization to relieve cellular oxidative stress [42,43]. Notably, peroxisome-engineering was shown to be substantially effective in enhancing protopanaxadiol; a significant increase in protopanaxadiol production (up to 4.1 ± 0.2 mg/L, *p* < 0.01) in CPX113436PPXP(pRS426-ADH2) was induced by ADH2 overexpression, which was ca. 8% higher than that in CPX113436PPXP without ADH2 overexpression (3.8 ± 0.5 mg/L, *p* < 0.05) and ca. 78% higher than that in the control non-engineered peroxisome WTPPX strains (2.3 ± 0.5 mg/L) (Figure 7b). Notably, dammarenediol II was not detected, indicating that dammarenediol II was completely transformed into protopanaxadiol and that ADH2 overexpression did not significantly affect WTPPXP and CPX1134PPXP.

## 4. Discussion

Most isoprenoids are lipophilic or hydrophobic, and their innate accumulation in the cell membrane highly influences diverse membrane functions of organisms, causing inhibitory effects on cell growth and cellular metabolisms. This inhibitory effect emerges as considerable hindrance in microbial bioprocess scale-ups in terms of growth reduction and decreased titer of target isoprenoid. In this study, as a proof of concept of organelle engineering in *S.cerevisiae* for enhancing protopanaxadiol production, we altered peroxisome biogenesis in *S.cerevisiae* by editing three genes encoding peroxins (Pex34p and Pex11p, and Atg36p), which are proteins involved in peroxisome proliferation. Furthermore, the protopanaxadiol biosynthesis pathway compartmentalization in peroxisomes significantly enhanced protopanaxadiol titer. Finally, the combination of peroxisome proliferation and pathway compartmentalization increased protopanaxadiol concentration to ca. 78% higher than that in the wild-type strain.

The high concentration of lipophilic natural products, which accumulates intracellularly in microbial hosts, can hardly be obtained by limited cellular storage capacity or cell cytotoxicity. A few studies have suggested that hydrophobic products, such as triacylglycerol [54] and carotenoids [55], are distributed into lipid droplets which serve as alternative storage depots. However, the complicate biosynthesis and maintenance of lipid storage is a major hurdle to the fulfillment of this attractive alternative [56]. The potential of overcoming limited cellular storages using peroxisomes has been confirmed through several studies [57,58]. In this study, the enhanced production of protopanaxadiol in the peroxisome-engineered strains was mainly owing to improved and stably maintained cellular membrane storage (increased size and copy number of peroxisomes) to accommodate protopanaxadiol (Figure 1). The expanded storage capacity could be easily observed through TEM and fluorescent microscopy; however, the causation of the observed improved titer of protopanaxadiol simply though the enlargement of storage capacity (peroxisomes) remains unclear. We hypothesize that as the proteins involved in MVA pathway are distributed in cytosol, endoplasmic reticulum (ER), and peroxisomes [16,59], more metabolic flux of precursor (such as farnesyl pyrophosphate (FPP)) might be redirected toward compartmentalized protopanaxadiol synthesis in peroxisomes. Although the current titers of protopanaxadiol in the peroxisome biogenesis-engineered strains developed in this study were lower than those reported in other studies [28], where metabolic engineering and synthetic biology strategies were intensively applied, we anticipate synergic effects of genetic combination of isoprenoid-enhancing modules (or gene manipulations) and peroxisome-biogenesis manipulation. For example, the MVA-pathway proteins [21] can be targeted into peroxisomes with increased size and copy number, which subsequently could enhance the supply of FPP for protopanaxadiol biosynthesis (Figure 5a) and consequently increase protopanaxadiol production in peroxisome-engineered *S.cerevisiae*. Furthermore, additional improvements can be obtained by introducing the many genetic and metabolic engineering interventions already established to facilitate isoprenoid production in yeast, including improving redox and pathway balancing [60], protein activity [61], and cofactor availability [62].

Even though this study serves as a pioneering milestone of “real/meaningful” engineering of peroxisomes, additional experimental evidence should be gathered in follow-up studies for wide application of peroxisome-biogenesis engineering for other isoprenoids. In conclusion, this method of peroxisome engineering could serve as an alternative strategy for the enhancement of other isoprenoids, and further experimental evidence should be gathered for the applicability of peroxisome biogenesis to diverse isoprenoids.

## Figures and Tables

**Table 1:** Strains used in this study.

Strains	Relevant Properties	Source or Reference
*Saccharomyces cerevisiae*		
CEN.PK2-1D	*MATa/α* *ura3-52 trp1-289 leu2-3_112 his3∆1 MAL2-8C SUC2*	This study
CEN-P11	CEN.PK2-1D, Δ*PEX11::P_TRP1_-TRP1-T_TRP1_*	This study
CEN-P30	CEN.PK2-1D, Δ*PEX30::P_TRP1_-TRP1-T_TRP1_*	This study
CEN-P5	CEN.PK2-1D, *TRP1::P_PGK1_-PEX5-T_CYC1_*	This study
CEN-P34-5	CEN-P34, *Leu2::P_PGK1_-PEX5-T_CYC1_*	This study
CPX34	CEN.PK2-1D, *TRP1::P_PGK1_-PEX34-T_CYC1_*	This study
CPX1134	CEN.PK2-1D, Δ*PEX11:: 3MYC-P_PGK1_-PEX34-T_CYC1_-3MYC*	This study
CPX36	CPX36, Δ*PEX11:: 3MYC-P_PGK1_-PEX34-T_CYC1_-3MYC*	This study
CPX113436	CPX36, Δ*PEX11:: 3MYC-P_PGK1_-PEX34-T_CYC1_-3MYC*	This study
WT-PG	CEN.PK2-1D, *POT1::P_POT1_-POT1-GFP-T_POT1_*	This study
CPX34-PG	CPX34, *POT1::P_POT1_-POT1-GFP-T_POT1_*	This study
CPX1134-PG	CPX1134, *POT1::P_POT1_-POT1-GFP-T_POT1_*	This study
CPX36-PG	CPX36, *POT1::P_POT1_-POT1-GFP-T_POT1_*	This study
CPX113436-PG	CPX113436, *POT1::P_POT1_-POT1-GFP-T_POT1_*	This study
WTDM	CEN.PK2-1D, *Leu2::P_GPD_-ERG9-T_CYC1_-P_PGK1_-ERG1pg-T_CYC1_*, *TRP1::P_TEF1_-DSpg-T_CYC1_-P_GPD_-tHMG1-T_CYC1_*	This study
WTDMP	CEN.PK2-1D, *Leu2::P_GPD_-ERG9-PTS1-T_CYC1_-P_PGK1_-ERG1pg-PTS1-T_CYC1_*, *TRP1::P_TEF1_-DS-PTS2-T_CYC_-P_GPD_-tHMG1-T_CYC1_*	This study
CPX1134DM	CPX1134, *Leu2::P_GPD_-ERG9-T_CYC1_-P_PGK1_-ERG1pg-T_CYC1_*, *TRP1::P_TEF1_-DSpg-T_CYC1_-P_GPD_-tHMG1-T_CYC1_*	This study
CPX1134DMP	CPX1134, *Leu2::P_GPD_-ERG9-PTS1-T_CYC1_-P_PGK1_-ERG1pg-PTS1-T_CYC1_*, *TRP1::P_TEF1_-DS-PTS2-T_CYC_-P_GPD_-tHMG1-T_CYC1_*	This study
WTPPXP	WTDM, *URA3:: P_PGK1_-PPDSpg-PTS1-T_CYC1_-P_TEF1_-CPRpg-T_CYC1_*	This study
CPX1134PPXP	CPX1134DMP, *URA3:: P_PGK1_-PPDSpg-PTS1-T_CYC1_-P_TEF1_-CPRpg-T_CYC1_*	This study
CPX113436PPXP	CPX1134DMP, Δ*ATG36::3MYC-P_URA3_-URA3-T_URA3_-3MYC*, *URA3:: P_PGK1_-PPDSpg-PTS1-T_CYC1_-P_TEF1_-CPRpg-T_CYC1_*	This study
*Escherichia coli*		
XL1-Blue	endA1 gyrA96(nal^R^) thi-1 recA1 relA1 lac glnV44 F’[::Tn10 proAB^+^ lacI^q^ Δ(lacZ)M15 Amy Cm^R^] hsdR17(r_K_^-^m_K_^+^)	Stratagene

**Table 2:** Plasmids used in this study.

Strains	Relevant Properties	Source or Reference
pRS424_GPD	YX-type shuttle vector, T7, lac, GPD promoter, 2micron, f1, pMB1 replicon, ampR, TRP1	ATCC 87357
pRS426_GPD	YX-type shuttle vector, T7, lac, PGK1 promoter, 2micron, f1, pMB1 replicon, ampR, URA3	ATCC 87359
pRS426_PGK1	pRS426-GPD, GPD promoter is replaced with PGK1 promoter, 2micron, f1, pMB1 replicon, ampR, URA3	This study
pRS424_GPD_ERG9	Constitutively expressed *ERG9* gene from *S.cerevisiae* CEN. PK2-1D	This study
pRS426_PGK1_ERG1	Constitutively expressed *ERG1* gene from *Panax ginseng*	This study
pRS424_GPD_DS	Constitutively expressed *DS* gene from *Panax ginseng*	This study
pRS424_GPD_PPDS	Constitutively expressed *PPDS* gene from *Panax ginseng*	This study
pRS424_GPD_ERG9_P1_	Constitutively expressed *ERG9* gene with PTS1 at C-terminal from *S.cerevisiae* CEN. PK2-1D	This study
pRS426_PGK1_ERG1p1	Constitutively expressed *ERG1* gene with PTS1 at C-terminal from *Panax ginseng*	This study
pRS424_GPD_DS_P1_	Constitutively expressed *DS* gene with PTS1 at C-terminal from *Panax ginseng*	This study
pRS424_GPD_DS_P2_	Constitutively expressed *DS* gene with PTS2 at N-terminal from *Panax ginseng*	This study
pRS424_GPD_PPDS_P1_	Constitutively expressed *PPDS* gene with PTS1 at C-terminal from *Panax ginseng*	This study
pRS424_GPD_EGFP	Constitutively expressed *EGFP* gene	This study
pRS424_GPD_EGFP_P1_	Constitutively expressed *EGFP* gene with PTS1 at C-terminal	This study
pRS424_GPD_ERG9_EGFPp1	Constitutively expressed *ERG9* and *EGFP* fusion gene with PTS1 at C-terminal from *S.cerevisiae* CEN. PK2-1D	This study
pRS424_GPD_ERG1_EGFPp1	Constitutively expressed *ERG1* and *EGFP* fusion gene with PTS1 at C-terminal from *Panax ginseng*	This study
pRS425_GPD_DS_mKOp1	Constitutively expressed *DS* and *mKO* fusion gene with PTS1 at C-terminal from *Panax ginseng*	This study
pRS425_GPD_DS_mKOp2	Constitutively expressed *DS* and *mKO* fusion gene with PTS2 at N-terminal from *Panax ginseng*	This study
pRS424_GPS_PPDS_EGFPp1	Constitutively expressed *PPDS* and *EGFP* fusion gene with PTS1 at C-terminal from *Panax ginseng*	This study
pCEV-G1	pSP-G1-type shuttle vector, TEF1 and PGK1 duel promoter, G418/kanamycin/neomycin resistance	Addgene #46813
pCEV-G1-TEF1-tHMG1	Constitutively expressed truncated *HMG1* gene from *S.cerevisiae* CEN. PK2-1D	This study
pCEV-G1-TEF1_DS	Constitutively expressed *DS* gene from *Panax ginseng* by TEF1 promoter	This study
pCEV-G1-TEF1_DS_P2_	Constitutively expressed *DS* gene with PTS2 at N-terminal from *Panax ginseng* by TEF1 promoter	This study
pCEV-G1-PGK1_PPDS	Constitutively expressed *PPDS* gene from *Panax ginseng* by PGK1 promoter	This study
pCEV-G1-PGK1_PPDS_P1_	Constitutively expressed *PPDS* gene with PTS1 at C-terminal from *Panax ginseng* by PGK1 promoter	This study
pCEV-G1-TEF1_CPR	Constitutively expressed *CPR* gene from *Panax ginseng* by TEF1 promoter	This study
pRS426-PGK1_ADH2	Constitutively expressed *ADH2* gene from *Saccharomyces cerevisiae* CEN PK2-1D by PGK1 promoter	This study
YIplac128	YI-type shuttle vector, lac promoter, pBR322 origin, ampR, LEU2	ATCC 87592
YIplac204	YI-type shuttle vector, lac promoter, pBR322 origin, ampR, TRP1	ATCC 87591
YIplac211	YI-type shuttle vector, lac promoter, pBR322 origin, ampR, URA3	ATCC 87593
YIplac128_ERG9	Constitutively expressed *ERG9* gene from *S.cerevisiae* CEN. PK2-1D with GDP promoter	This study
YIplac128_ERG9_P1_	Constitutively expressed *ERG9* gene with PTS1 at C-terminal from *S.cerevisiae* CEN. PK2-1D with GDP promoter	This study
YIplac128_ERG9_ERG1	Constitutively expressed ERG9 gene from *S.cerevisiae* CEN. PK2-1D with GDP promoter and *ERG1* gene from *Panax ginseng* with PGK1 promoter	This study
YIplac128_ERG9p1_ERG1p1	Constitutively expressed ERG9 gene with PTS1 at C-terminal from *S.cerevisiae* CEN. PK2-1D with GDP promoter and *ERG1* gene with PTS1 at C-terminal from *Panax ginseng* with PGK1 promoter	This study
YIplac204_tHMG1	Constitutively expressed *truncated HMG1* gene from *S.cerevisiae* CEN. PK2-1D with TEF1 promoter	This study
YIplac204_tHMG1_DS	Constitutively expressed *truncated HMG1* from *S.cerevisiae* CEN. PK2-1D and *DS* gene from *Panax ginseng* with TEF1 promoters	This study
YIplac204_tHMG1_DS_P2_	Constitutively expressed *truncated HMG1* from *S.cerevisiae* CEN. PK2-1D and *DS* gene with PTS2 at N-terminal from *Panax ginseng* with TEF1 promoters	This study
YIplac211_CPR	Constitutively expressed *CPR* gene from *Panax ginseng* with TEF1 promoter	This study
YIplac211_CPR _PPDS	Constitutively expressed *CPR* and *PPDS* gene from *Panax ginseng* with TEF1 and PGK1 promoters	This study
YIplac211_CPR _PPDS_P1_	Constitutively expressed *CPR* gene and *PPDS* gene with PTS1 at C-terminal from *Panax ginseng* with TEF1 and PGK1 promoters	This study
YIplac128_tHMG1	Constitutively expressed *truncated HMG1* gene from *S.cerevisiae* CEN. PK2-1D with TEF1 promoter	This study
pUC57_URA blast	Cloning vector for *E.coli*, URA selectable marker cassette with 3Myc site at both N-terminal and C-terminal for integration in Yeast genome	KITECH

## Data Availability

Not applicable.

## Supplementary Materials

The following supporting information can be downloaded at: https://www.mdpi.com/article/10.3390/microorganisms10030650/s1, Table S1: Primers used in this study.
