# Supplementary material for: Organelle Engineering in Yeast: Enhanced Production of Protopanaxadiol through Manipulation of Peroxisome Proliferation in Saccharomyces cerevisiae
Source: Microorganisms. 2022 Mar 18;10(3):650. doi: 10.3390/microorganisms10030650 (PMC8950469; doi:10.3390/microorganisms10030650)
Supplement: Supplementary file 1 [file microorganisms-10-00650-s001.zip › microorganisms-1633422-supplementary.pdf]

microorganisms

Supplementary materials

Table S1. Primers used in this study.

Gene	Sequence (5′ to 3′)	Enzyme site
tHMG1	F: CGGCGGCCGCAAAATGTCTATTCCAGAAACTC<br>R: GCACTAGTTTATTTAGAAGTGTCAACAA	*NotI*<br>*SpeI*
ERG9	F: GCGGGATCCAAAATGGAAAGCTATTACAAT<br>R: CGCTCGAGGCTCACGCTCTGGTAAAGTG	*BamHI*<br>*XhoI*
ERG9-PTS1	F: GCGGGATCCAAAATGGAAAGCTATTACAAT<br>R: CGCCTCGAGGCTCACAACTTAGACGCTCTGTGTAAAGTG	*BamHI*<br>*XhoI*
ERG1	F: GCGGGATCCAAAATGTTTACTTTGAGGAGG<br>R: CGGCTAGCCGTTATGGGCGGGGATTT	*BamHI*<br>*NheI*
ERG1-PTS1	F: GCGGGATCCAAAATGTTTACTTTGAGGAGG<br>R: CGGCTAGCCGTTACAACTTAGATGGGCGGGGATTT	*BamHI*<br>*NheI*
DS	F: CGCGCGGCCGCAAAATGGCAAAGCAAAAGGG<br>R: CGACTAGTGCTTAAATTTTCAGTTGTTGGTGC	*NotI*<br>*SpeI*
DS-PTS1	F: CGCGCGGCCGCAAAATGGCAAAGCAAAAGGG<br>R: CGACTAGTGCTTACAACTTAGAAATTTTCAGTTGTTGGTGC	*NotI*<br>*SpeI*
DS-PTS2	F: GCGCGGCCGCAAAATGGCAAAGAGACTACAAAGTATCAAGGA TCATTTGCAAAAGGG<br>R: CGACTAGTGCTTAAATTTTCAGTTGTTGGTGC	*NotI*<br>*SpeI*
PPDS	F: GCGGGATCCAAAATGGTGTTATTTTTCTCCC<br>R: CGCCCGGGGCTTAGTTGTGAGGATGCAAAT	*BamHI*<br>*SmaI*
PPDS-PTS1	F: GCGGGATCCAAAATGGTGTTATTTTTCTCCC<br>R: CGCCCGGGGCTTACAACTTAGAGTTGTGAGGATGCAAAT	*BamHI*<br>*SmaI*
CPR	F: CGGCGGCCGCAAAATGGCTAAAGTGTCTCCCTTCGA<br>R: GCACTAGTTTACCATACATCACGCA	*NotI*<br>*SpeI*
ADH2	F: CGGCGGCCGCAAAATGTCTATTCCAGAAACTC<br>R: GCACTAGTTTATTTAGAAGTGTCAACAA	*NotI*<br>*SpeI*
P_{GPD}-*ERG9*-T_{CYC1}	F: GCGCATGCAGTTTATCATTATCAATACTC<br>R: GCTCTAGAGCGGCCGCAAATTAAAGC	*SphI*<br>*XbaI*
P_{PGK1}-*ERG1*-T_{CYC1}	F: GCCTGCAGAGTTTATCATTATCAATACTC<br>R: CGCGTCGACGGCCGCAAATTAAAGC	*PstI*<br>*SalI*
P_{TEF1}-*tHMG1*-T_{ADH1}<br>P_{TEF1}-*DS*-T_{ADH11}<br>P_{TEF1}-*CPR*-T_{ADH1}	F: CGCGGTACCCACACACCATAGCTTC<br>R: CGCGGTACCCACTAGGAGCGACC	*KpnI*<br>*KpnI*
P_{PGK1}-*PPDS*-T_{CYC1}	F: GCGAATTCGAAGTACCTTCAAAGAAT<br>R: GCTCTAGACTTCGAGCGTCCCAA	*EcoRI*<br>*XbaI*
URA3	F: TGTTCGTACCACCAAGGAATTACTGGAGTTAGTTGAAGCATTAG GTCCCAAGTTTATCATTATCAATACTC<br>R: AGACCACATCATCCACGGTTCTATACTGTTGACCCAATGCGTCTC CCTTGATTATCATGACATTAACCTAT	
LEU2	F: ACGTTGGTCAAGAAATCACAGCCGAAGCCATTAAGGTTCTTAA AGCTATTAGTTTATCATTATCAATACTC<br>R: ATGGCCTTACCTTCTTCAGGCAAGTTCAATGACAATTTCAACATC ATTGCATTATCATGACATTAACCTAT	
TRP1	F: ACCAAGAGTTCCTCGGTTTGCCAGTTATTAAAAGACTCGTATTT CCAAAAAGTTTATCATTATCAATACTC	

	R:<br>AACACCAATAACGCCATTTAATCTAAGCGCATCACCAACATTTTC<br>TGGCGATTATCATGACATTAACC TAT
ΔATG36	F: TGTATTCAGGGCTTAAAATACTAAAATTTGGTGGTCAGTACAGT<br>TCATTAGACGTTGTAAAACGACGGCC<br>R: CTCACATCATTGAATGACTTTAATCTATGGAAGTCTAGCTCAAAT<br>TCCTCCACACAGGAAACAGCTATGACC
POT1-EGFP	F: GTATGTGTATCGGTACTGGTATGGGTGCCGCCGCCATCTTTATTA<br>AAGAACGGATCCCCGGGTTAATTAA<br>R:<br>ATATGAGCATAATAAAAAGGGAGAATATTAACTATTATCAAGTAT<br>TAAAAGAATTCGAGCTCGTTTAAAC
